# Underreporting of inferior vena cava filter characteristics in diagnostic radiology reports: a call for standardization

**DOI:** 10.1186/s42155-025-00576-5

**Published:** 2025-10-28

**Authors:** Gavin Wu, David S. Shin, Neha Naidoo, Spencer B. Lewis, Jeffrey Forris Beecham Chick, Eric J. Monroe, Anthony N. Hage, Mina S. Makary

**Affiliations:** 1 Division of Vascular and Interventional Radiology, Department of Radiology, The Ohio State University Wexner Medical Center, 395 W 12 Avenue, Columbus, OH 43210 USA; 2 Division of Vascular and Interventional Radiology, Department of Radiology, University of Southern California, 975 Zonal Avenue, Los Angeles, CA 90033 USA; 3 Section of Interventional Radiology, Department of Radiology, University of Washington, Seattle, WA 98195 USA; 4 Radiology Specialists of the Northwest, 5050 NE Hoyt Street, Portland, OR 97213 USA; 5 Section of Vascular and Interventional Radiology, Department of Radiology, University of Wisconsin, 600 Highland Avenue, Madison, WI 53792 USA; 6 Department of Radiology, Section of Vascular and Interventional Radiology, 1 Cooper Plaza, Camden, NJ 08103 USA

To the Editor

The use of retrievable inferior vena cava (IVC) filters has grown significantly in the United States, with more than 24 distinct filter types currently in clinical use (Fig. 1) [1]. While IVC filter retrieval is strongly recommended once the indication for filtration resolves, retrieval rates remain under 50% nationally [2]. Diagnostic imaging, particularly radiography and CT, offers a chance to flag filters that may be suitable for removal. However, the documentation of IVC filter type and retrievability in diagnostic radiology reports remains inconsistent and poorly characterized.

**Fig. 1:**
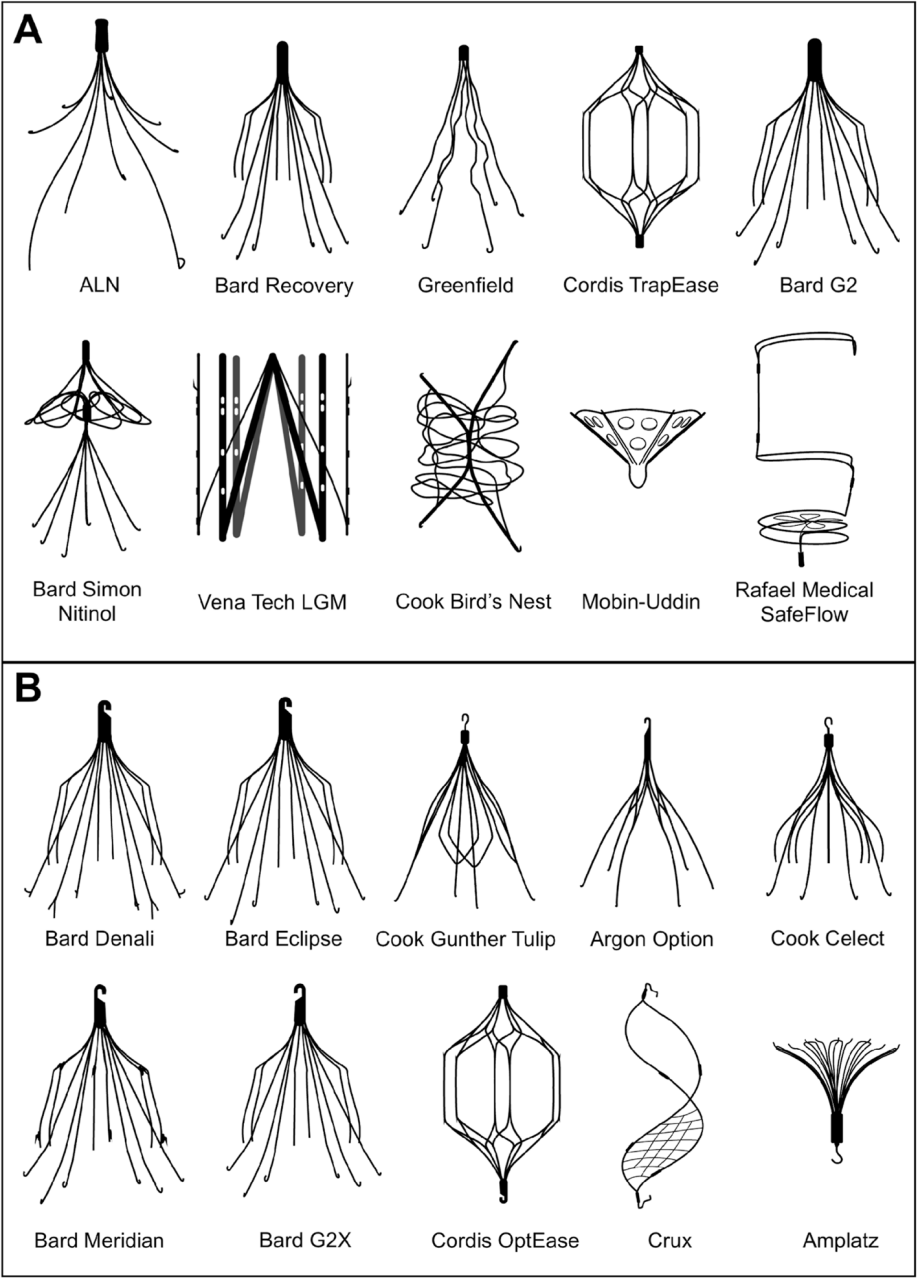
Schematic diagram of inferior vena cava filter models. **A** Permanent filters. **B** Retrievable filters

We retrospectively reviewed 10,217 radiography, CT, and PET-CT studies that referenced “IVC filter” or “inferior vena cava filter” between 2000 and 2019. Procedural, ultrasound, MRI, and other non-qualifying exams were excluded. Retrievability (permanent vs retrievable) and manufacturer model were recorded when mentioned. Of these studies, only 17 reports (0.2%) mentioned retrievability (Fig. 2) and 138 reports (1.4%) identified a specific manufacturer model, of which nearly half of the latter were misidentified (Fig. 3). Errors included generic labeling of all conical filters as Greenfield and confusion between filters with similar morphology. Although manufacturer-specific details may not always be expected in diagnostic reports, these misclassifications underscore the lack of familiarity with IVC filter anatomy among general radiologists.

**Fig. 2:**
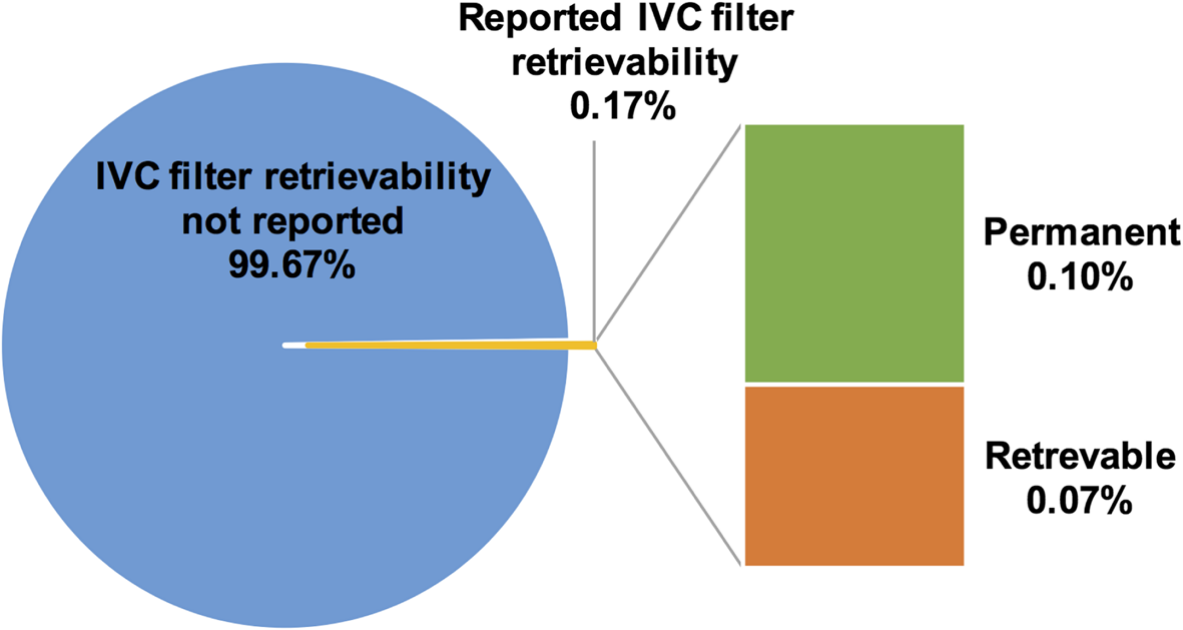
Frequency of IVC filter retrievability documentation in diagnostic radiology reports

**Fig. 3:**
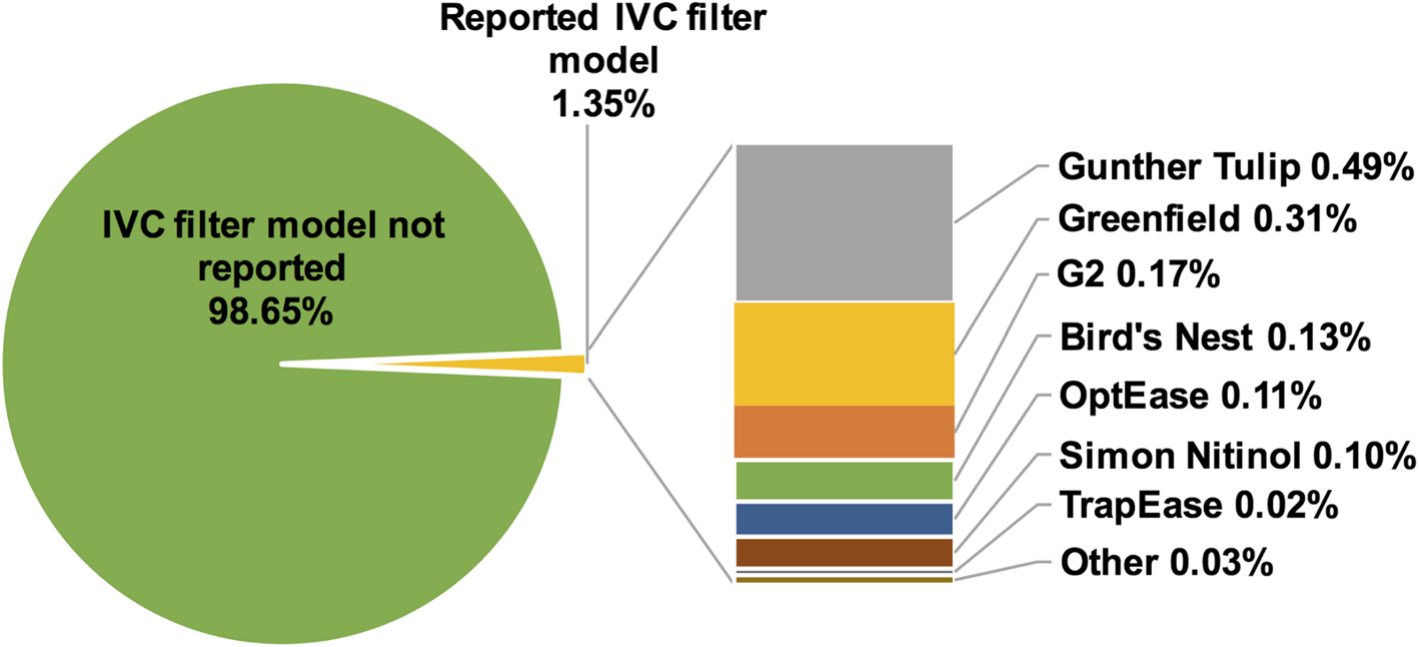
Frequency and accuracy of IVC filter manufacturer model reporting

Limitations of this study include its retrospective design, reliance on keyword search, and the exclusion of procedural reports, which are expected to include more detailed device descriptions. We also acknowledge that retrievability is multifactorial and cannot be assessed on imaging alone.

Nonetheless, the results highlight the need for more standardized reporting practices, as missing or inaccurate documentation of IVC filter features can lead to delayed retrieval and increased complication risk. For instance, mislabeling a retrievable filter as permanent may lead to missed retrieval and expose patients to filter fracture, migration, or caval thrombosis [3]. Future efforts should focus on integrating standard language or optional macros to improve documentation and create consistency across institutions. Indeed, structured reporting tools for IVC filters have previously shown promise in identifying abnormal filters [4]. In addition, diagnostic imaging provides an opportunity to re-engage patients lost to follow-up after IVC filter placement [5]. As these patients often reenter care through unrelated imaging, standardized filter reporting practices may provide a route to return these patients to the necessary care required for their IVC filters.

In conclusion, although the reporting of IVC filter retrievability and manufacturer model in diagnostic radiology remains limited, it represents an opportunity for standardization and clinical impact. Diagnostic radiologists can play a key role in identifying patients with potentially retrievable filters, and simple measures such as noting filter type and retrievability may support appropriate management and improve outcomes.

## Data Availability

All data generated or analysed during this study are included in this published article.

## Abbreviations

IVC: Inferior vena cava
